# The Salts of Strontium

**Published:** 1892-06

**Authors:** 


### THE SALTS OF STRONTIUM.

A good deal is being written in the journals about the therapeutics of strontium. Professor Laborde, for some time has been collecting experimental and clinical evidence as to the physiological action and therapeutic effect of the salts of this metal, and his conclusion has been that the drug possesses powers for good not sufficiently recognized. Other well known authorities, such as Brunton, Dujardin-Beaumetz, Germain See and others, practically agree with Laborde. Clinically the bromide of strontium is found exceedingly useful in those nervous disorders in which the bromides of sodium and potassium cannot be satisfactorily employed. It does not disturb the stomach functions, while appetite and digestion are even said to be improved by it. The lactate of strontium seems to possess decided virtue in the treatment of albuminuria. Although not a diuretic, it does diminish the amount of albumen excreted and leads to an improvement in all the collateral symptoms of albuminuria. Albumen reappears whenever the drug is suspended and disappears again as soon as it is resumed. Different observers agree that the salt is indicated in the "parenchymatous nephritis of gouty and rheumatic subjects, as well as in puerperal and post-puerperal albuminuria." In five cases of various origins, nephritic, cardiac, etc., treated by Dujardin-Beaumetz, he reduced the amount of albumen as much as fifty per cent. within one to four days. This gentleman concluded:

In lactate of strontium we possess an invaluable agent, the action of which is at the same time certain and inoffensive.

